# Home-based self-help telerehabilitation of the upper limb assisted by an electromyography-driven wrist/hand exoneuromusculoskeleton after stroke

**DOI:** 10.1186/s12984-021-00930-3

**Published:** 2021-09-15

**Authors:** Chingyi Nam, Bingbing Zhang, Tszying Chow, Fuqiang Ye, Yanhuan Huang, Ziqi Guo, Waiming Li, Wei Rong, Xiaoling Hu, Waisang Poon

**Affiliations:** 1 Department of Biomedical Engineering, The Hong Kong Polytechnic University, Hong Kong, China; 2 Department of Surgery, Prince of Wales Hospital, The Chinese University of Hong Kong, Hong Kong, China; 3 The Hong Kong Polytechnic University Shenzhen Research Institute, Shenzhen, 518034 China

**Keywords:** Stroke, Robot, Rehabilitation, Home training, Telerehabilitation

## Abstract

**Background:**

Most stroke survivors have sustained upper limb impairment in their distal joints. An electromyography (EMG)-driven wrist/hand exoneuromusculoskeleton (WH-ENMS) was developed previously. The present study investigated the feasibility of a home-based self-help telerehabilitation program assisted by the aforementioned EMG-driven WH-ENMS and its rehabilitation effects after stroke.

**Methods:**

Persons with chronic stroke (n = 11) were recruited in a single-group trial. The training progress, including the training frequency and duration, was telemonitored. The clinical outcomes were evaluated using the Fugl–Meyer Assessment (FMA), Action Research Arm Test (ARAT), Wolf Motor Function Test (WMFT), Motor Functional Independence Measure (FIM), and Modified Ashworth Scale (MAS). Improvement in muscle coordination was investigated in terms of the EMG activation level and the Co-contraction Index (CI) of the target muscles, including the abductor pollicis brevis (APB), flexor carpi radialis-flexor digitorum (FCR-FD), extensor carpi ulnaris-extensor digitorum (ECU-ED), biceps brachii (BIC), and triceps brachii (TRI). The movement smoothness and compensatory trunk movement were evaluated in terms of the following two kinematic parameters: number of movement units (NMUs) and maximal trunk displacement (MTD). The above evaluations were conducted before and after the training.

**Results:**

All of the participants completed the home-based program with an intensity of 63.0 ± 1.90 (mean ± SD) min/session and 3.73 ± 0.75 (mean ± SD) sessions/week. After the training, motor improvements in the entire upper limb were found, as indicated by the significant improvements (P < 0.05) in the FMA, ARAT, WMFT, and MAS; significant decreases (P < 0.05) in the EMG activation levels of the APB and FCR-FD; significant decreases (P < 0.05) in the CI of the ECU–ED/FCR–FD, ECU–ED/BIC, FCR–FD/APB, FCR–FD/BIC, FCR–FD/TRI, APB/BIC and BIC/TRI muscle pairs; and significant reductions (P < 0.05) in the NMUs and MTD.

**Conclusions:**

The results suggested that the home-based self-help telerehabilitation program assisted by EMG-driven WH-ENMS is feasible and effective for improving the motor function of the paretic upper limb after stroke.

*Trial registration* ClinicalTrials.gov. NCT03752775; Date of registration: November 20, 2018.

**Supplementary Information:**

The online version contains supplementary material available at 10.1186/s12984-021-00930-3.

## Introduction

Most patients with stroke who are discharged home from inpatient poststroke rehabilitation have residual motor impairment of the upper limb, especially in the distal joints (i.e., the wrist and the fingers), which greatly inhibits their ability to perform activities of daily living (ADLs) [1, 2]. Although the traditional viewpoint on poststroke rehabilitation suggested that significant motor recovery mainly occurs in the first 6 months after the onset of a stroke (i.e., acute and subacute periods) [3], more recent studies have indicated that significant motor improvements could also be achieved in the chronic period after stroke through physical training as long as such training is as intensive as the one provided to inpatients [4, 5]. Continuous and regular physical therapy is required to improve the wrist/hand function of outpatients with chronic stroke [6]. The restoration of limb function after stroke depends on intensive and repetitive training of the paralyzed limb [7, 8] with maximized voluntary motor effort [9, 10] and minimized compensatory motions in close-to-normal muscular coordination [10, 11]. However, the provision of effective wrist/hand rehabilitation services for outpatients with chronic stroke is insufficient in the current healthcare system in the world.

In most cases, outpatients have limited access to wrist/hand treatments with the necessary training intensity [12, 13] because of resource constraints due to factors such as an expanding stroke population and a lack of professionals worldwide [14, 15], as well as other difficulties such as commuting [2] to the outpatient services in day hospitals, and the restriction of social distancing during the COVID-19 pandemic. Home-based telerehabilitation with minimum assistance and remote supervision by professionals (i.e., self-help operation) is a promising approach for sustaining of physical treatment after discharge and enhancing the accessibility of rehabilitation resources to improve the wrist/hand motor functions of discharged patients [16–18].

However, few studies have focused on techniques for effective self-help upper limb rehabilitation, especially for distal joints [16]. Currently, most studies on home-based telerehabilitation have been based on virtual reality (VR) techniques because home-based VR training is more convenient for and accessible to outpatients than conventional therapy in a clinic or day hospital [16, 19]. Nevertheless, those systems focus on assessment or monitoring of limb performance rather than providing the necessary physical assistance for the patients to achieve the desired movements [16, 20–24]. Rehabilitation robots have been developed to provide mechanical assistance that mimics physical support from a therapist in conventional therapy; these robots can alleviate the labor-intensive aspects of hands-on physical therapy by performing repetitive therapeutic tasks intensively under the supervision of a therapist [15], and these robotic therapies for distal joints have been reported to be effective for improving upper limb motor function [1, 25]. However, the majority of the existing rehabilitation robots are heavy, have complex mechanical designs, and require large power supplies, large physical spaces in conventional environments (e.g., clinic), and close professional supervision, which are significant deterrents to their use by patients independently at home [15, 26].

Furthermore, using robot alone has a limitation in directly activating the desired muscle groups because the target muscles of patients with stroke usually cooperate with compensatory motions from other muscular activities [27]. Compensatory motions from the trunk and the proximal joints, i.e., abnormal motor synergies, are commonly observed in most persons with chronic stroke when they attempt to reach an object or orient their hand to grasp an object [28]. When neuromuscular electrical stimulation (NMES) combined with robotic therapy, the robotic assistance could provide sensorimotor experiences with precise kinematics to realize the desired movements [29], and NMES could activate the target muscles and reduce compensation from alternative muscle synergies [30]. Thus, the combined NMES-robot treatment has been suggested to facilitate close-to-normal muscular coordination with reduced compensation motions, and it has yielded more effective rehabilitation outcomes than upper limb rehabilitation treatments that use only NMES or only robots [31]. Electromyography (EMG) of the paralyzed limb to indicate voluntary intention to integrate voluntary motor effort during practice has been recommended for optimizing therapeutic outcomes [32]. EMG-driven training systems have yielded superior improvements in motor functions with longer sustainability than those with passive limb motions [33], especially for voluntary motor control of the upper limb. Therefore, EMG-driven NMES-robot therapy for home-based self-help training is desirable for effective wrist/hand rehabilitation for outpatients with chronic stroke.

**Fig. 1:**
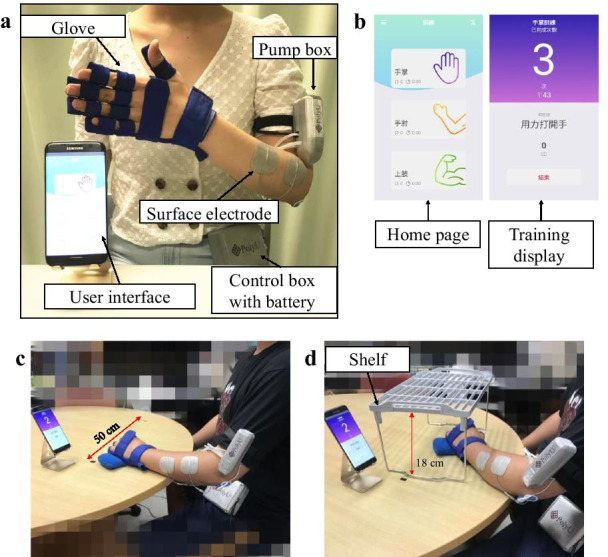
Experimental and training set up of the EMG-driven WH-ENMS. **a** Photograph of the EMG-driven WH-ENMS and **b** the screen captures of the interface. The training set up in a session assisted by the EMG-driven WH-ENMS and configuration of **c** the horizontal task and **d** vertical task

A novel EMG-driven exoneuromusculoskeleton (ENMS) for self-help upper limb rehabilitation after stroke was developed recently by our team [34, 35]. Taking the advantages of exoskeleton, pneumatic muscle, and NMES, the developed system is lightweight, compact, and has low power consumption. The system can assist the extension and flexion of the elbow, wrist, and finger joints under voluntary effort control through EMG. The system consists of an elbow module and a wrist/hand module that can work collectively or separately. The wrist/hand module can work independently as an EMG-driven wrist/hand ENMS (WH-ENMS) to assist wrist/hand movements during training (Fig. 1). The rehabilitation effects of the EMG-driven ENMS have been investigated in 15 participants with chronic stroke [35], followed by a 20-session training program in a neurorehabilitation laboratory, where the participants completed the training independently with the system after they received a tutorial session and three guided training sessions (including practicing device operation and training setup). The participants exhibited significant improvements in voluntary motor control and muscle coordination of the paretic limb after the EMG-driven ENMS-assisted upper limb training [35]. No safety problems were reported by either the experiment operators or the participants throughout the study period. The system offers the possibility of home-based self-help wrist/hand training for discharged patients with chronic stroke. However, the feasibility of using the EMG-driven WH-ENMS for self-help upper limb training and its rehabilitation effects in a home setting had not been investigated.

Therefore, in this study, we aimed to determine the feasibility of home-based self-help training assisted by the EMG-driven WH-ENMS on outpatients with chronic stroke and investigate its rehabilitation effects. Our hypothesis was that the participants who received the home-based self-help telerehabilitation training assisted by the EMG-driven WH-ENMS would obtain motor improvements in the distal joints, better muscle coordination of the paretic upper limb, and reduce compensatory movements when performing limb tasks.

## Methods

A single-group trial was conducted on discharged patients with chronic stroke (n = 11) who underwent a home-based self-help telerehabilitation program consisting of 20 sessions of wrist/hand training assisted by the EMG-driven WH-ENMS. The training outcomes were evaluated through clinical assessments, EMG evaluations and kinematic analysis.

### EMG-driven WH-ENMS

The EMG-driven WH-ENMS used in this study is shown in Fig. 1a, b. The system can be worn on the paretic upper limb and assist a stroke survivor in performing phasic wrist/hand coordinated movements, namely (1) wrist extension with the hand open and (2) wrist flexion with the hand close.

The system consists of a wearable glove with a textile bracing on the hand, a pump box mounted on the upper arm and a control box carried on the waist. A rechargeable 12-V Li-ion battery inside the control box can support continuous system usage for 4 h. The wearable glove with the embedded musculoskeletal hand comprises five pneumatic finger muscles and a three-dimensional printed exoskeletal connector fixed on the palm side. The pneumatic muscles can provide extension torque to individual digits during inflation, and it deflated when the valve is opened. Each pneumatic muscle can generate a maximal extension torque of 0.1 Nm across the MCP joint of a finger when its inner pressure reaches 96 kPa [35]. Two-channel NMES was applied to the wrist/finger extensors (i.e., the extensor carpi ulnaris (ECU) and the extensor digitorum (ED)) and flexors (i.e., the flexor carpi radialis (FCR) and the flexor digitorum (FD)) to assist wrist/finger extension and flexion, respectively, through two pairs of reusable surface electrodes (5 × 5 cm^2^, PALS Neurostimulation Electrodes, Axelgaard Manufacturing Co., Ltd., Fallbrook, CA, USA). The muscles of the ECU and ED and the muscles of the FCR and FD were treated as an ECU-ED muscle union and a FCR -FD muscle union, respectively, for both NMES and EMG detection in this study due to the close anatomical proximity between the FCR and FD muscles and between the ECU and ED muscles [36]. The NMES outputs were square pulses with a constant amplitude of 70 V, stimulation frequency of 40 Hz, and a manually adjustable pulse width in the range of 0–300 µs (with a threshold pulse width to evoke maximal muscle contraction) [35]. A pair of surface electrodes was used for both EMG detection and NMES delivery to a target muscle union, and these electrodes were located in the common area of the motor point of the two muscle bellies of the muscle union [35]. A reference electrode (2 × 3 cm^2^, Blue Sensor N, Ambu Inc., Ballerup, Denmark) was attached to the skin surface of the olecranon to attenuate the common mode noise.

To facilitate the phasic wrist/hand movements, the ECU-ED and FCR -FD muscle unions were used as voluntary neuromuscular drives to control mechanical assistance and NMES assistance from the system. EMG-triggered control was adopted in this study [35]. Three times the standard deviation (SD) above the EMG baseline in the resting state was set as the threshold level in each motion phase. In the “wrist extension with the hand open” phase, once the EMG activation level of the ECU-ED reached the preset threshold, NMES was applied to the ECU-ED and mechanical extension torque was provided to the fingers by the inflated pneumatic finger muscles to assist joint extension of the wrist and the fingers throughout the motion phase. In the “wrist flexion with the hand close” phase, as soon as the EMG activation level of the FCR -FD reached the preset threshold, the pneumatic finger muscles were deflated passively and NMES was applied to the FCR-FD to assist joint flexion of the wrist and the fingers throughout the motion phase. The residual voluntary effort from the finger flexors of the paretic limb can facilitate the release of air from the pneumatic muscles during deflation. A detailed description of the assistive control can be found in our previous study on the design of the EMG-driven ENMS for poststroke rehabilitation [35].

The captured EMG signals were first amplified 1000 times (preamplifier: INA 333; Texas Instruments Inc., Dallas, TX, USA) and filtered from 10 to 500 Hz. These amplified and filtered signals were then sampled using an analog-to-digital converter (AD73360, Analog Devices Inc., Norwood, MA, USA) with a sampling frequency of 1000 Hz for each EMG channel. After digitization, the EMG signals were full-wave rectified and moving-averaged with 100-ms window to obtain the EMG activation level during real-time control [35].

Real-time control and wireless communication between the control unit and a mobile android application (app) were achieved on a smartphone by using a microprocessor and a Bluetooth module (Bluetooth HC-05, JMoon Technologies., New Delhi, India). The app was designed as the user interface to communicate with the system and provide visual indications to patients. The designed app emphasized patient convenience, ease of use, large-font instructions with clear options, and straightforward navigation for user-independent operation. A patient can start or stop a training session by simply tapping the button in the app interface. In addition, an emergency stop button of the battery power supply to the control box was also provided as the single switch of the control box to simplify system operation, and to shut down the system in case of an emergency. The training data was recorded by the app and transmitted to the server computer located in the laboratory through a mobile network of 3G or above automatically after a user had exited the app, for telemonitoring the training progress.

### Home-based training program

#### Subject recruitment

**Fig. 2:**
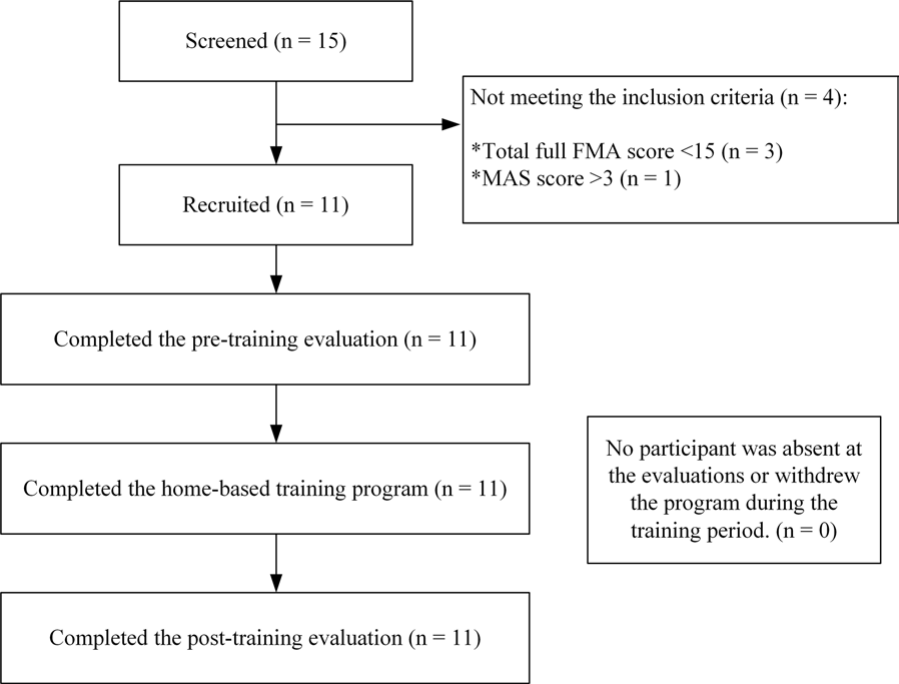
The Consolidated Standards of Reporting Trials flowchart of the experimental design

After obtaining the ethical approval from the Human Subjects Ethics Subcommittee of the Hong Kong Polytechnic University, a total of 15 participants from local districts were screened. Eleven participants with chronic stroke were recruited in this study. The inclusion criteria included (1) at least 1 year after the onset of a singular and unilateral brain lesion due to stroke, (2) discharge from hospital, (3) the spasticity at the elbow, wrist and fingers were ≤ 3 as measured by the Modified Ashworth Scale (MAS) [37], (4) motor impairments in the affected upper limb ranging from severe to moderate according to the Fugl-Meyer Assessment (FMA; 15 < FMA < 45, with a maximal score of 66 for the upper limb) [38], (5) no visual deficits and the ability to understand and follow simple instructions, as assessed by the Mini-Mental State Examination (MMSE > 21) [39], (6) presence of detectable voluntary EMG signals from the driving muscle on the affected side (three times the SD above the EMG baseline), (7) presence of active ROM of shoulder from 30° to 80° flexion, (8) presence of passive ROM of wrist from 45° extension to 60° flexion, and the ability of the MCP finger joints to be passively extended to 170°, (9) fulfillment of minimum living environment requirements, including a table measuring at least with 60 × 40 cm^2^ as the training space, a bridge chair without wheels, and a 3G or above mobile network coverage at home. Subjects were excluded if they (1) were currently pregnant, (2) were epileptic or (3) had an implanted pacemaker. Before the commencement of the clinical trial, written informed consent was obtained from each participant. Figure 2 shows the Consolidated Standards of Reporting Trials flowchart of the experimental design.

#### Interventions

The rehabilitation program consisted of a pre-training tutorial and a 20-session upper limb training assisted by the EMG-driven WH-ENMS (at least 60 min/session), with the intensity of 3–5 sessions/week, within 7 consecutive weeks, and with no more than 1 session/day.

**Fig. 3:**
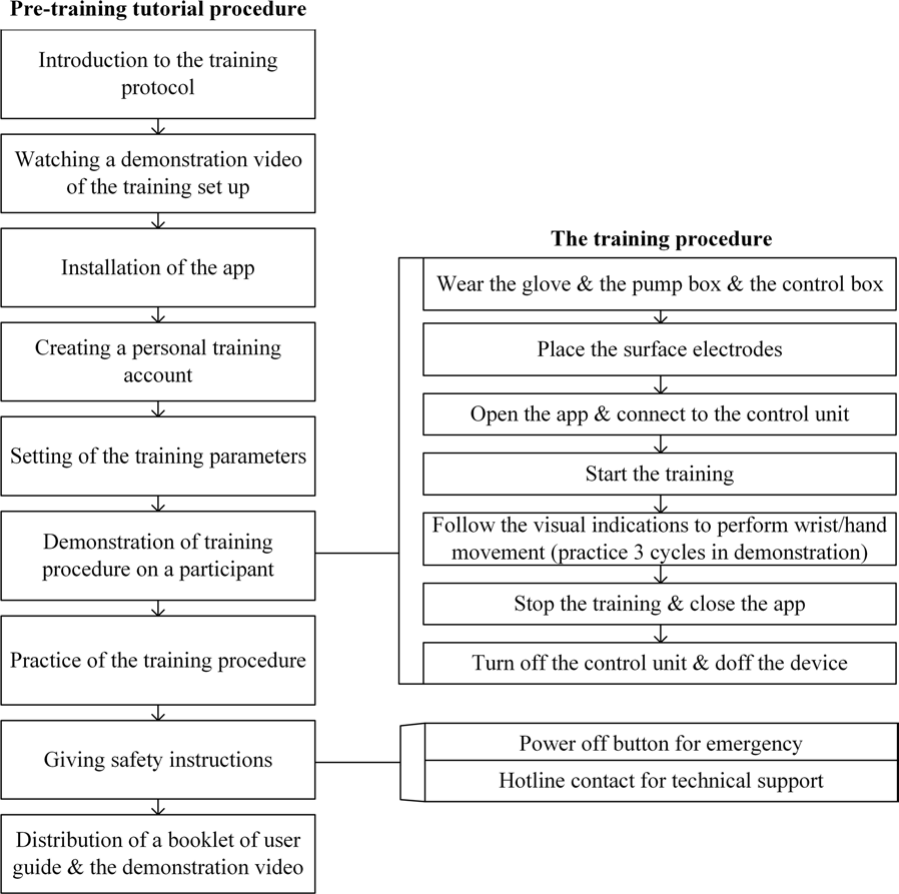
Pre-training tutorial procedure

Before the training, a pre-training tutorial lasting 30 to 45 min on donning and doffing the system, device operation and the training protocol was provided to each participant. The procedure of the pre-training tutorial is shown in Fig. 3. If any participant was not using an Android smartphone or if they did not have a smartphone, they were lent an Android smartphone with the developed app until they completed the training program. Regarding the setting of training parameters, the operator set the training parameters i.e., the EMG triggering levels of the driving muscle unions, maximum inner pressure of the wrist/hand module, and the applied pulse width of NMES for individual participants. These parameters remained fixed throughout the 20-session training. The maximal inner pressure of the wrist/hand module was set to < 100 kPa during training, to ensure stability of the pneumatic muscles under repeated inflations and deflations. When placing surface electrodes and reference electrode on the participant’s arm, the operator marked those positions on the participant’s skin and instructed the participants to retain these markings until the last session. Once the electrode position markings faded, the participants were required to remark them with a marker pen or a ballpoint pen. Moreover, safety instructions were provided during the tutorial.

The training started within 3 days after the tutorial. In each training session, the participants were required to sit at a table and to maintain a vertical distance of 30–40 cm between the table surface and their shoulder (Fig. 1c, d). The smartphone with the app was positioned on the table and placed in front of the participant at a horizontal distance of 30–60 cm. The participants were required to follow the visual indications displayed on the smartphone screen and perform the repetitive limb tasks assisted by the EMG-driven WH-ENMS on the paretic limb. The participants were then required to perform 30-min horizontal task and 30-min vertical task (Table 1) at their natural speed. A 10-min rest between two consecutive tasks was allowed to prevent muscle fatigue during training sessions.

**Table 1:** Descriptions of the required upper limb movements for motion tasks

Task	Description
Horizontal	A participant was instructed to grasp a sponge that was placed on one side of a table near the paretic side of the participant, transport the sponge 50 cm horizontally (i.e., horizontal transportation phase I), release it, grasp it again, move it back to the starting point (i.e., horizontal transportation phase II), and release it
Vertical	A participant was instructed to grasp the sponge on the table surface, lift it up (i.e., vertical transportation phase I), place it on the top of the shelf (with a vertical distance of 18 cm), grasp it again, place it back on the table surface (i.e., vertical transportation phase II), and release it

**Fig. 4:**
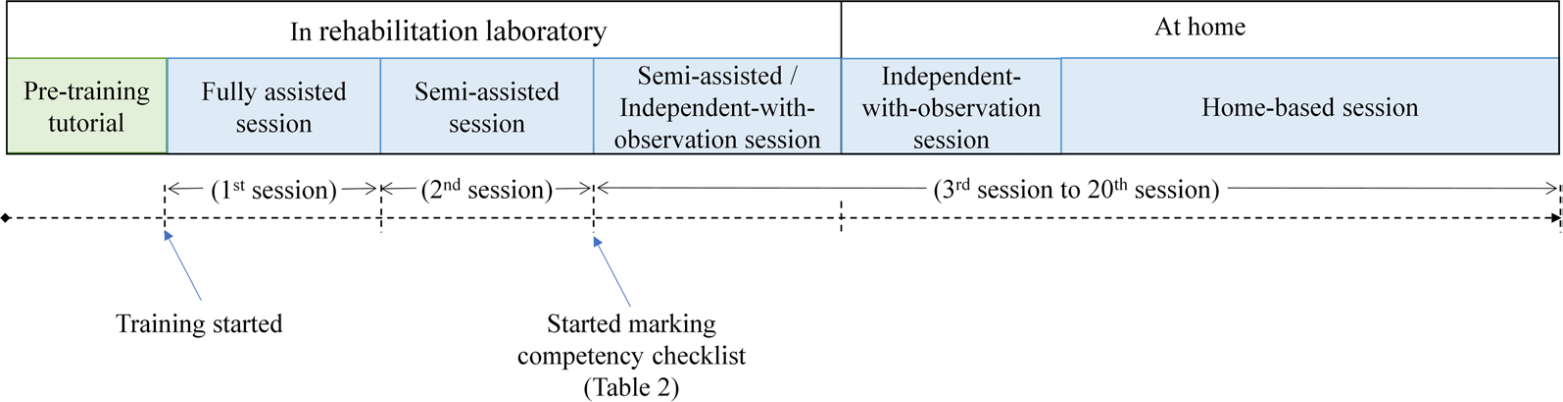
Timeline of the EMG-driven WH-ENMS-assisted home-based self-help upper limb training program

In this study, the first three training sessions were conducted in the rehabilitation laboratory and were supervised by the experiment operator to reinforce and test the competency of the participants in performing home-based self-help training. In the supervised sessions, nearby professional assistance was provided at varying levels, namely (1) fully assisted, where the operator supported the participants from the training setup and supervised the entire training process in the first session; (2) semi-assisted, where the participants completed the session mainly by themselves, with minimum assistance from the operator in the second session; and (3) independent-with-observation, where the participants completed the training session independently under close observation by the operator. An additional semi-assisted session was offered to participants who were not ready for the independent-with-observation session. During the third session, the operator marked the competency checklist (Table 2) to assess the participant’s competency in conducting home-based training with the system. Once the participants correctly demonstrated all of the technical items listed on the competency checklist, they were required to start the home-based training for the remaining sessions. If a participant had a personal caregiver, the caregiver was also invited to attend the tutorial and training sessions and allowed to provide assistance with the setup. Figure 4 shows the timeline of the EMG-driven WH-ENMS-assisted home-based self-help upper limb training program.

**Table 2:** Competency checklist

Is the participant able to demonstrate the following abilities appropriately when using the system?	Yes	No
Don and doff the device		
Turn on and turn off the device		
Login and logout of the personal training account		
Connect the app and the device		
Attach the electrodes onto their arm		
Start and stop the training		
Recharge the device		
**Is the participant able to demonstrate the following abilities appropriately during the training?**	**Yes**	**No**
Safely and correctly perform all of the training tasks in a session		
Place two markers at a horizontal distance of 50 cm on the table for the horizontal task		
Place the shelf with the correct setting for the vertical tasks		
Understand the safety precautions and warnings associated with device usage during training		
If the participant joins with a caregiver, the caregiver is allowed to assist with the training setup If the answer is “No” for any item, the participant is deemed to be not competent to perform the home-based training with the system. Otherwise, the participant is deemed as competent Experiment operator: ______________________ Signature: _____________________ Date: Number of current session:

#### Logistics Management of Home-based Training

Prior to the commencement of the home-based training, the operator first arranged the training schedules for the remaining sessions agreed by a participant, and subsequently delivered the system with a battery charger and training props (e.g., a sponge and a shelf) to the participants’ homes. In the first home-based session, the operator visited the participants’ homes to inspect environmental safety (e.g., training space and nearby electricity) and then observed the entire session to ensure consistency with the session in the laboratory. The participants were required to complete the remaining sessions at home without on-site professional supervision (home-based sessions). The participants could change the training schedule occasionally by informing the operator 1 day in advance so that the operator could arrange a make-up session for the participant without violating the protocol intensity, i.e., 3–5 sessions/week. Finally, the participants were required to return the system to the research team during their post-training evaluation visit to the laboratory, which was scheduled 1 day after the last training session.

The logistics management data recorded by the developed app, including the number of completed wrist/hand movement cycles, total training time, and completion date/time point of a session, were automatically transmitted via a mobile network to the server computer located in the laboratory. The data were reviewed by the operator through an encrypted network which required the operator to enter a pre-set login account and password [40], for telemonitoring the training progress of the participants. The experiment operator remotely monitored the completion of each home-based session based on the participants’ commitment to the training schedule. Once a missing session was found, the operators contacted the participant by telephone to schedule a make-up session.

If there was any technical issue during the home-based sessions, the participants were able to inform the experiment operator immediately through a phone call or a message. A backup system was prepared for each participant and used to replace the malfunctioning system. The participants were required to return the malfunctioning system to the laboratory and have it replaced with the backup system in 1 working day.

### Evaluation of training outcomes

Clinical assessments, EMG measurements, and kinematic measurements were adopted to investigate the rehabilitation outcomes of the home-based self-help upper limb training assisted by the EMG-driven WH-ENMS. All evaluations were conducted at the laboratory. All of the participants underwent evaluations just before the pre-training tutorial (i.e., pre-training evaluation) and 1 day after the last training session (i.e., post-training evaluation).

#### Clinical assessments for functional evaluation

In this study, clinical assessments were used to evaluate the motor functional improvements of each participant and were conducted by a training-blinded assessor. The adopted clinical assessments included (1) the FMA that the full score is 66 for the upper limb assessment, which has been sub-scaled into shoulder/elbow (42/66) and wrist/hand (24/66) [41], was adopted to measure the motor functional impairment in voluntary limb movements; (2) the Action Research Arm Test (ARAT) [42], was applied to evaluate the upper limb voluntary functions with a focus on the finger activities; (3) the Wolf Motor Function Test (WMFT) [43], was used to assess the functional ability and motion speed of the upper limb in daily tasks; (4) the Motor Functional Independence Measure (FIM) [44], was adopted to evaluate the basic quality of participant’s ADLs; and (5) the MAS [37] on the flexors related to the elbow, wrist, and fingers, was used to measure the poststroke spasticity at the related joints.

#### Evaluation of muscular coordination in the upper limb by EMG

**Fig. 5:**
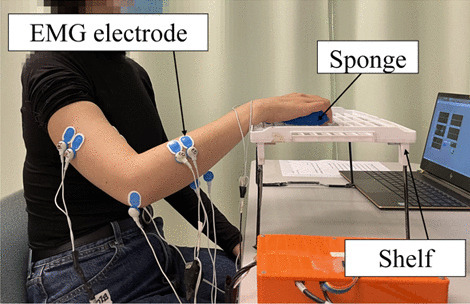
Configuration of EMG recording during the bare arm test

Two EMG parameters were used to obtain quantitative information on the muscle activation and coordination patterns of the paretic limb. They were (1) the EMG activation level of each target muscle and (2) the EMG co-contraction index (CI) between muscle pairs [45, 46]. More details of the EMG measurement are described in Additional file 1: Appendix S1 Part A with experimental setup shown in Fig. 5.

#### Evaluation of movement smoothness and compensatory trunk movement

**Fig. 6:**
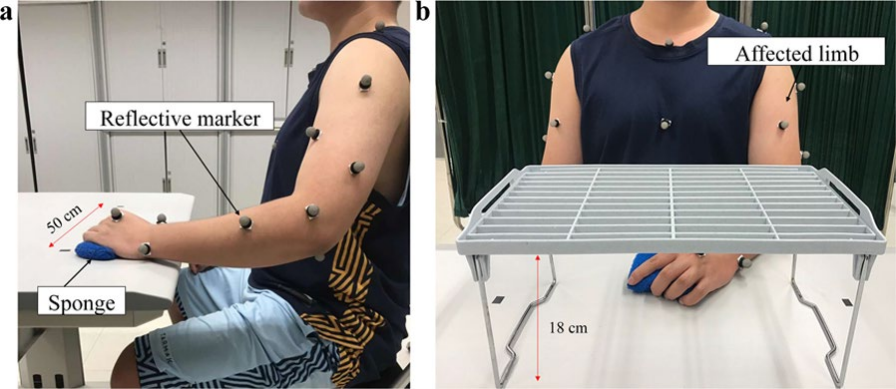
Experimental setup for **a** the horizontal task and **b** vertical task during three-dimensional motion capturing

Three-dimensional motion analysis was performed to quantify the kinematic performance of the upper limb, and this was used as an outcome measure to evaluate impaired movement after stroke [47, 48]. Two kinematic parameters, (1) number of movement units (NMUs) and (2) maximal trunk displacement (MTD) [49, 50], were adopted to obtain quantitative information about movement smoothness and compensatory trunk movement. More details of the kinematic measurement are shown in Additional file 1: Appendix S1 Part B with experimental setup shown in Fig. 6.

### Statistics

The normality tests on the clinical scores, EMG parameters and kinematic parameters were evaluated using the Shapiro test with a significance level of 0.05 [51, 52]. The MAS exhibited significance in the normality test (P < 0.05), and the FMA, ARAT, WMFT, FIM, EMG parameters and kinematic parameters exhibited nonsignificant probabilities (P > 0.05). Wilcoxon’s signed rank test was performed on the MAS using a paired comparison of the scores before and after the training. Paired-sample t test was used to detect the differences in the FMA, ARAT, WMFT, FIM, EMG data and kinematic data before and after the training. The primary outcome of this study was the FMA. The other clinical scores, EMG parameters, and kinematic parameters were secondary outcomes. The statistically significant level of 0.05 was used for all tests in this study. The significance levels at 0.01 and 0.001 were indicated as well.

## Results

All of the recruited participants (n = 11) completed the home-based self-help upper limb training assisted by the EMG-driven WH-ENMS. The demographic data of the participants are shown in Table 3. The demographic characteristics of each participant are shown in Table S1A in Additional file 2: Appendix S2.

**Table 3:** Demographic characteristics of the patients with stroke recruited for the home-based self-help upper limb training program (n = 11)

Subject No.	Gender Female/Male	Stroke Types Hemorrhagic/ Ischemic	Side of Hemiparesis Left/ Right	Age (years) Mean ± SD	Years after onset of stroke Mean ± SD
11	6/5	5/6	8/3	57.6 ± 13.2	13.4 ± 10.4

### Independency in self-help upper limb training at home

In this study, all of the participants were determined as having sufficient competence to perform the home-based training after the third session, and no additional semi-assisted session was required by them. Six of the participants completed the training at home independently, whereas the others completed the training with partial assistance from their caregivers at home. Four of the participants conducted and completed the home-based self-help training during the COVID-19 pandemic in Hong Kong. No adverse event (e.g., pain or injury) during or after the training was reported by either the experiment operators or the participants throughout the study period.

According to the logistic data captured using the app, the average training frequency in the 20-session training was 3.73 ± 0.75 (mean ± SD) sessions/week, with a range of 3–5 sessions/week. Variability was observed in the duration of the home-based training, with an average session duration of 63.0 ± 1.90 (mean ± SD) min/session (ranging from 60 to 66 min/session), and average complete wrist/hand movement cycles per session of 116 ± 8.72 (mean ± SD). More details of the recorded logistic data are shown in Table S1B in Additional file 2: Appendix S2. The earliest and latest training times were 07:00 and 22:00, respectively. The peak training hours were between 14:00 to 16:00 and 19:00 to 21:00, with more than 80% of the home-based sessions being conducted during those periods. Five participants encountered technical issues during the home-based training sessions, among which three were related to broken leads because of excessive pulling on the leads and two were related to air leakage from the musculoskeleton hand due to hard and repeated squeezing of the pneumatic muscles during inflation. These problems were solved within 1 working day, and the related components were further reinforced. Furthermore, the malfunctioning systems were also replaced with backup systems within 1 working day.

### Training outcomes of the home-based self-help program

#### Clinical assessments

**Fig. 7:**
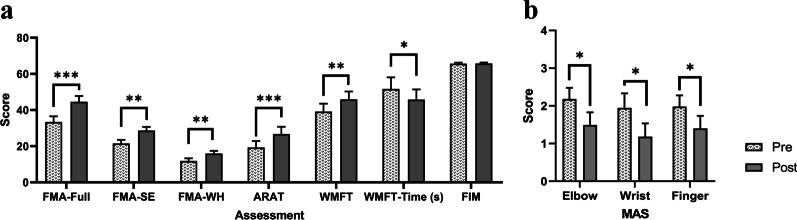
The measured clinical scores of the **a** FMA, ARAT, WMFT and FIM, and **b** the MAS before (Pre) and after (Post) the training represented by means and standard errors. Significance levels are indicated by * (P ≤ 0.05), ** (P ≤ 0.01), and *** (P ≤ 0.001)

Table 4 lists all of the clinical scores measured in this study (i.e., the means and 95% confidence intervals of each clinical assessment, together with the paired-sample *t* test/ Wilcoxon’s signed rank test probabilities with effect sizes (EFs) for evaluations before and after the training). The comparison of the clinical scores measured before and after the training is shown in Fig. 7. In Fig. 7a, a significant increase was found after the training in the FMA full score, FMA shoulder/elbow, FMA wrist/hand, ARAT, WMFT score, and WMFT time (P < 0.05, paired-sample *t* test). In Fig. 7b, a significant decrease in the MAS scores was observed after the training at the elbow, the wrist and the fingers (P < 0.05, Wilcoxon’s signed rank test). The individual outcomes of clinical scores of each participant are shown in Table S1C in Additional file 2: Appendix S2.

**Table 4:** Means and 95% confidence intervals of each measurement in the clinical assessments and the probabilities and estimated effect sizes in the statistical analyses. Differences with statistical significance are denoted by * (P ≤ 0.05), ** (P ≤ 0.01), and *** (P ≤ 0.001)

Evaluation	Pre	Post	Change ($+/-$)	Paired-sample *t* test	
	Mean (95% Confidence interval**)**			P-value	Cohen's *d*
FMA					
Full Score	33.4 (30.2 to 39.7)	44.5 (41.3 to 51.0)	$+$ 11.2 (7.53 to 14.8)	< 0.001***	1.84
Shoulder/Elbow	21.5 (19.6 to 25.4)	28.6 (26.6 to 32.7)	$+$ 7.10 (3.78 to 10.4)	0.002**	1.29
Wrist/Hand	11.8 (10.3 to 14.8)	15.9 (14.4 to 18.9)	$+$ 4.10 (2.18 to 6.00)	0.002**	1.29
ARAT	19.3 (15.7 to 26.5)	26.7 (22.7 to 34.8)	$+$ 7.45 (4.09 to 10.8)	0.001***	1.34
WMFT					
Score	39.2 (34.8 to 47.9)	45.9 (41.6 to 54.6)	$+$ 6.72 (3.23 to 10.2)	0.003**	1.16
Time	51.6 (45.2 to 64.5)	45.7 (40.1 to 57.0)	− 5.89 (− 10.7 to − 1.12)	0.033*	− 0.75
FIM	65.6 (65.1 to 66.8)	65.7 (65.2 to 66.9)	$+$ 0.09 (− 0.09 to 0.27)	0.341	0.30

MAS				Wilcoxon’s signed ranks test	
				P-value	r
Elbow	2.18 (1.59 to 2.77)	1.49 (0.82 to 2.17)	− 0.69 (− 1.16 to − 0.22)	0.026*	− 0.67
Wrist	1.95 (1.17 to 2.72)	1.18 (0.48 to 1.89)	− 0.76 (− 1.35 to − 0.17)	0.026*	− 0.67
Finger	1.98 (1.39 to 2.58)	1.40 (0.73 to 2.07)	− 0.58 (− 0.98 to − 0.18)	0.024*	− 0.68

#### EMG parameters

**Fig. 8:**
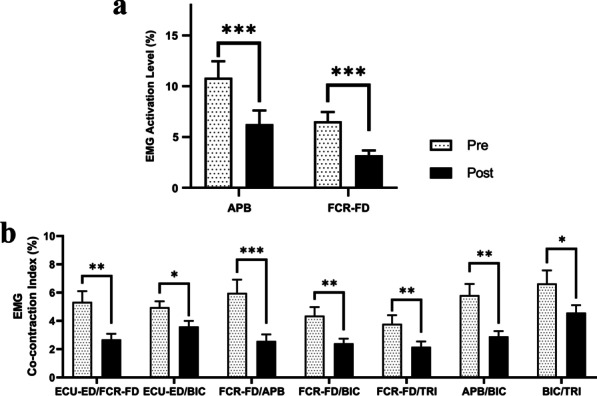
**a** EMG activation levels of the APB and FCR–FD during the bare hand evaluations, and **b** the EMG CI between the FCR–FD and ECU–ED, ECU–ED and BIC, FCR–FD and APB, FCR–FD and BIC, FCR–FD and TRI, APB and BIC, and BIC and TRI during the bare hand evaluations before (Pre) and after (Post) the training represented by means and standard errors. Significance levels are indicated by * (P ≤ 0.05), ** (P ≤ 0.01), and *** (P ≤ 0.001)

Table 5 lists the measured EMG parameters (i.e., the means and 95% confidence intervals of each clinical assessment, together with the paired-sample *t* test probabilities with EFs of the evaluation before and after the training). Figure 8 illustrates the EMG parameters (i.e., the normalized EMG activation level and the normalized CI) that were statistically significant before and after the training. A significant decrease in EMG activation level was detected in the APB and FCR-FD (P < 0.05) after the training (Fig. 8a). A significant reduction of CI in the muscle pairs of ECU-ED/FCR-FD, ECU-ED/BIC, FCR-FD/APB, FCR-FD/BIC, FCR-FD/TRI, APB/BIC and BIC/TRI (P < 0.05) was found after the training (Fig. 8b). No significant increase or decrease was observed in the EMG parameters of the other target muscles and muscle pairs.

**Table 5:** Means and 95% confidence intervals of each measurement in the EMG parameters as well as the probabilities and estimated effect sizes in the statistical analyses. Differences with statistical significance are denoted by * (P ≤ 0.05), ** (P ≤ 0.01), and *** (P ≤ 0.001)

EMG parameters	Pre	Post	Change ($+/-$)	Paired-sample *t* test	
	**Mean (95% Confidence interval) (%)**			P-value	Cohen's *d*
Normalized EMG activation level					
APB	10.8 (7.61 to 14.1)	6.25 (3.60 to 8.91)	− 4.59 (− 6.95 to − 2.23)	0.001***	− 0.83
ECU-ED	9.84 (7.45 to 12.2)	7.44 (5.20 to 9.68)	− 2.40 (− 5.78 to 0.98)	0.17	− 0.30
FCR-FD	6.52 (4.73 to 8.32)	3.21 (2.26 to 4.16)	− 3.31 (− 4.72 to − 1.90)	< 0.001***	− 1.00
BIC	7.71 (5.92 to 9.49)	6.12 (4.89 to 7.36)	− 1.58 (− 3.45 to 0.28)	0.11	− 0.36
TRI	7.05 (3.89 to 10.20)	5.77 (3.32 to 8.23)	− 1.27 (− 4.81 to 2.26)	0.48	− 0.15
Normalized CI					
APB/ ECU-ED	7.19 (4.65 to 9.72)	4.94 (2.56 to 7.32)	− 2.25 (− 5.49 to − 0.99)	0.18	− 0.30
APB/ FCR-FD	5.98 (4.10 to 7.86)	2.58 (1.64 to 3.51)	− 3.41 (− 5.25 to − 1.57)	0.001***	− 0.79
APB/BIC	5.82 (4.24 to 7.40)	2.91 (2.17 to 3.64)	− 2.91 (− 4.65 to − 1.18)	0.003**	− 0.72
APB/ TRI	6.04 (3.16 to 8.91)	4.56 (2.26 to 6.85)	− 1.48 (− 4.59 to 1.63)	0.35	− 0.20
ECU-ED/FCR-FD	5.34 (3.83 to 6.86)	2.69 (1.89 to 3.48)	− 2.65 (− 4.14 to − 1.17)	0.002**	− 0.76
ECU-ED/BIC	4.97 (4.14 to 5.81)	3.60 (2.81 to 4.40)	− 1.37 (− 2.48 to − 0.26)	0.022*	− 0.53
ECU-ED/TRI	4.18 (3.09 to 5.26)	3.88 (2.39 to 5.37)	− 0.30 (− 1.73 to 1.14)	0.684	− 0.09
FCR-FD/BIC	4.37 (3.15 to 5.59)	2.42 (1.75 to 3.08)	− 1.95 (− 3.04 to − 0.87)	0.002**	− 0.77
FCR-FD/ TRI	3.80 (2.59 to 5.01)	2.17 (1.42 to 2.92)	− 1.62 (− 2.67 to − 0.58)	0.005**	− 0.67
BIC/TRI	6.64 (4.80 to 8.49)	4.59 (3.54 to 5.63)	− 2.06 (− 3.92 to − 0.19)	0.039*	− 0.47

#### Kinematic parameters

**Fig. 9:**
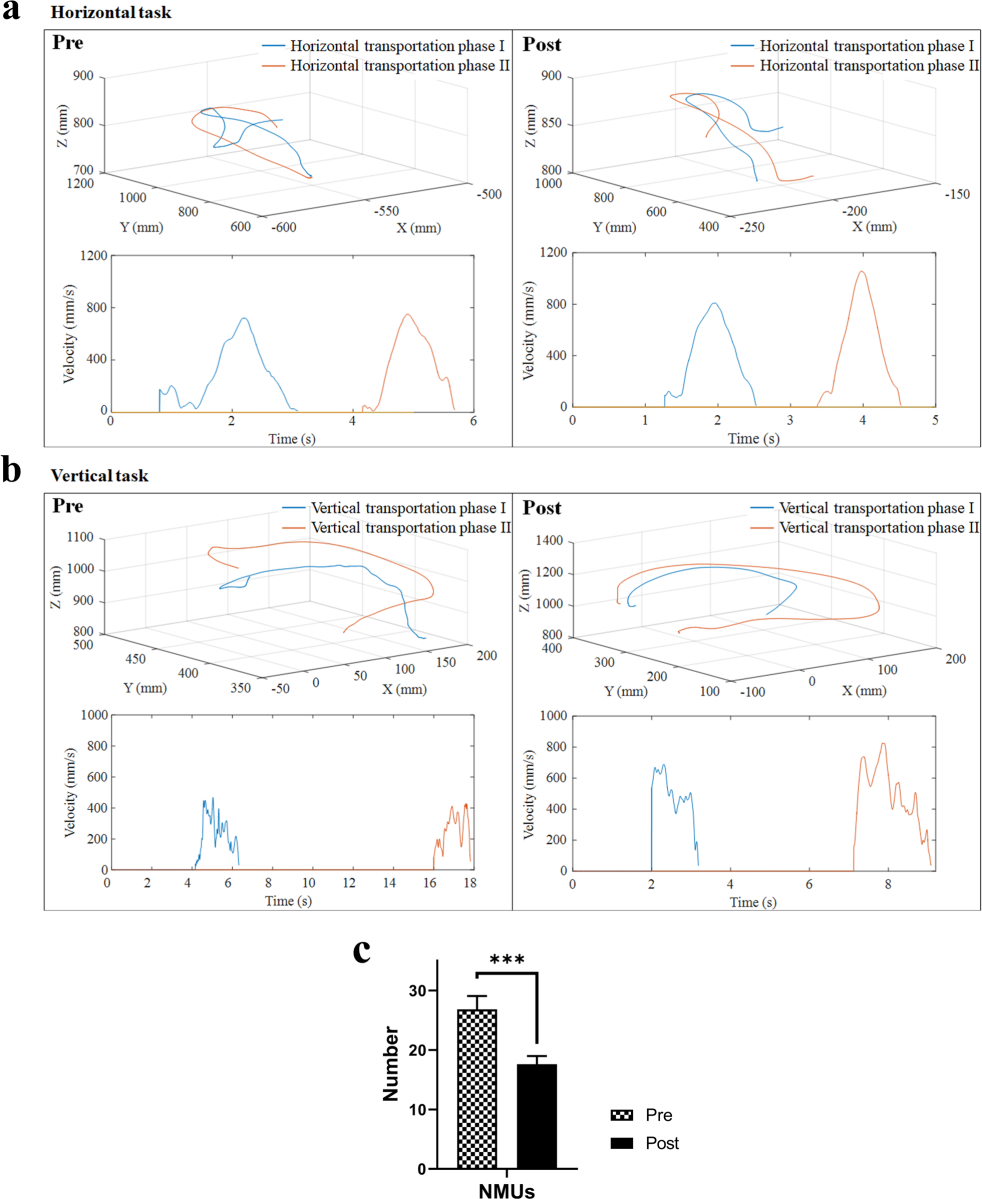
Representative measured trajectory of the hand marker during the transport phases in **a** the horizontal task and **b** vertical task for a participant, and the related velocity profiles of the trial before and after training. **c** The NMUs before (Pre) and after (Post) the training represented in terms of the mean and standard error. Significance levels are indicated by *** (P ≤ 0.001)

**Fig. 10:**
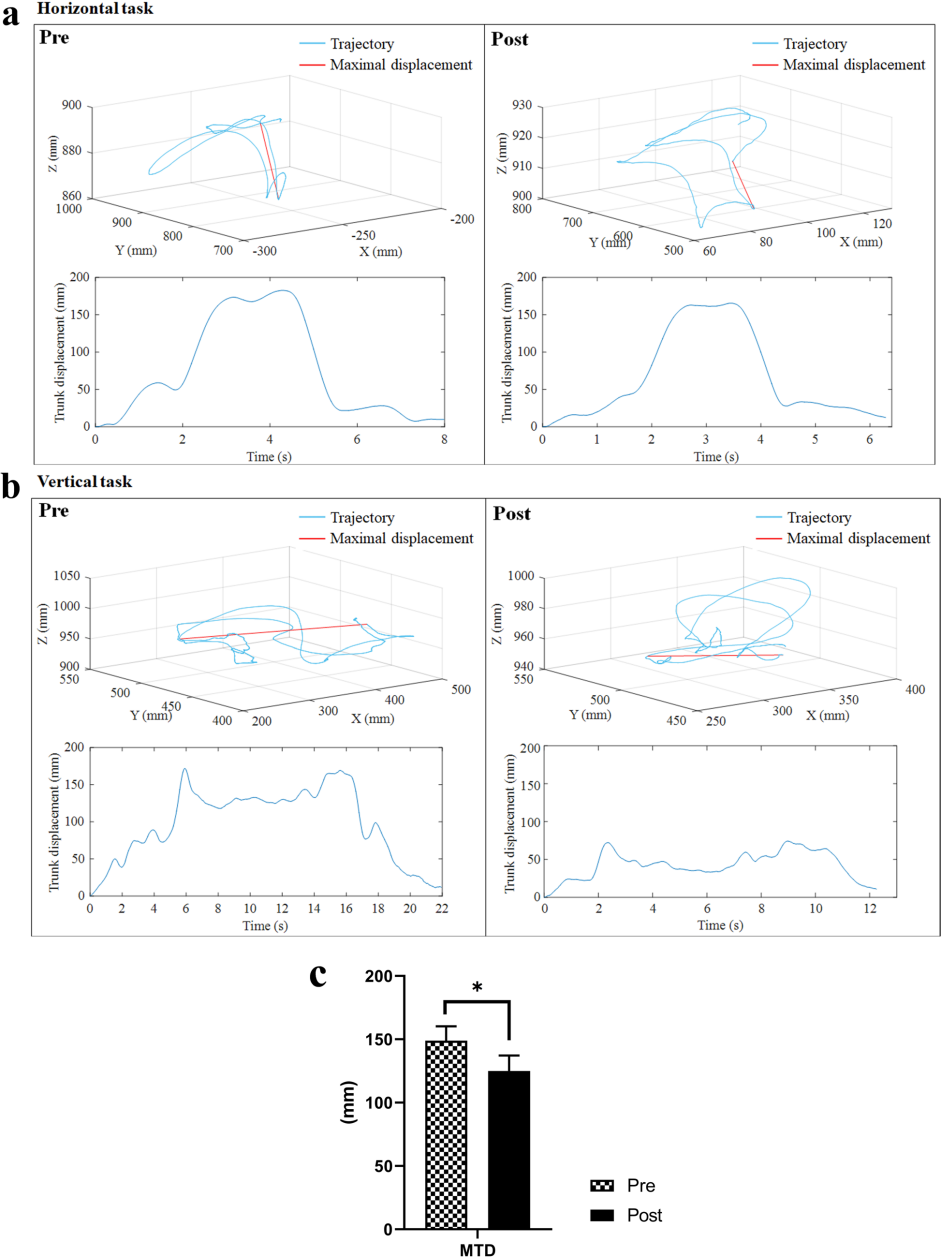
Representative measured trajectory of the thorax marker over the entire trial for **a** the horizontal task and **b** vertical task for a participant, and the related displacement profiles in the trial before and after the training. **c** The MTD before (Pre) and after (Post) the training represented in terms of the mean and standard error. Significance levels are indicated by * (P ≤ 0.05)

The measured kinematic parameters are listed in Table 6 (i.e., the means and 95% confidence intervals of each clinical assessment, together with the paired-sample *t* test probabilities with EFs of the evaluation before and after the training). Figure 9a, b shows the representative velocity profiles of the horizontal and vertical tasks before and after the training of a participant with respect to the three-dimensional trajectory of the hand marker during the transport phases. A significant decrease in NMUs was observed after the training (Fig. 9c; P < 0.05). Figure 10a, b illustrates the representative displacement profiles of a participant during horizontal and vertical tasks before and after the training with respect to the three-dimensional trajectory of the thorax marker throughout the entire trial. A significant reduction in MTD was found after the training (Fig. 10c; P < 0.05).

**Table 6:** Means and 95% confidence intervals of each measurement in the kinematic parameters as well as the probabilities and estimated effect sizes in the statistical analyses

Kinematic parameters	Pre	Post	Change ($+/-$)	Paired-sample *t* test	
	Mean (95% Confidence interval)			P-value	Cohen's *d*
NMUs	26.8 (22.3 to 31.3)	17.6 (14.8 to 20.4)	− 9.23 (− 13.0 to − 5.50)	< 0.001***	− 1.06
MTD	149 (126 to 172)	125 (101 to 149)	− 23.9 (− 42.1 to − 5.70)	0.016*	− 0.56

Differences with statistical significance are denoted by * (P ≤ 0.05) and *** (P ≤ 0.001)

## Discussion

The results of this study support the hypothesis that it is feasible to use EMG-driven WH-ENMS to support the home-based self-help upper limb training of discharged participants with chronic stroke. After 20-session training, motor function improvements associated with improved clinical scores, EMG parameters, and kinematic parameters were observed in all of the participants.

### Training outcomes of the home-based self-help program

#### Clinical scores

The significant increase in the FMA (shoulder/elbow and wrist/hand) score indicated an improvement in voluntary motor control of the entire upper limb after the training, especially on the joint stability and ROMs at the related joints on the paretic limb (Fig. 7a). The ARAT was adopted mainly to evaluate motor functions with hand tasks and focused on the finger functions, including holding/releasing objects in different shapes, sizes and weights. The significant increase in the ARAT score indicated the improvements in the hand function of the participants when handling different objects and coordination of the fingers for fine precision grasping (Fig. 7a). The increased WMFT scores suggested an overall improvement in the entire upper limb and increased ability to perform daily activities (Fig. 7a). The reduced time for conducting WMFT tasks (WMFT time) implied an improvement in movement efficiency and improved muscle coordination in the upper limb (Fig. 7a).

A significant improvement in the ability to perform daily activities was observed (as manifested in the increased WMFT scores and decreased WMFT time), but the improvement in the functional use of ADLs was not confirmed by the FIM scores. It indicated that the regained motor functions might not be translated into the functional use of the paretic limb to perform ADLs, even though the ability in the performance of daily tasks increased after the training. This was probably due to the two following characteristics of patients with chronic stroke: (1) learned nonuse could have become a habit, and the limb may not be used in functional activities although the individual has ability to move it [28], and (2) the unaffected limb attempts to execute all of the motor actions required for daily living [53]. It is suggested that outpatient rehabilitation should start as early as possible to minimize the development of learned nonuse and facilitate that the motor gains could translate into the functional use of the affected limb in ADLs.

The significant decrease in MAS score at the flexors of the elbow indicated that the spasticity of the elbow joint was reduced (Fig. 7b). The significantly reduced MAS scores at the flexors of the wrist and fingers indicated a release of spasticity of the distal joints (Fig. 7b). The reduced MAS score reflected a superior control over synergic muscle activity and a decrease in compensatory muscular activity [28, 54]. The decrease in the MAS scores of the elbow, wrist, and finger joints implied improved muscle coordination and joint stability of the proximal and distal joints during arm reaching, which was consistent with the observations in the FMA.

#### EMG parameters

A reduction in excessive muscle activities and improved muscle coordination was revealed by the decrease in the EMG activation levels at the flexors of the distal joints, co-contractions between the antagonist muscle pairs related to the wrist/hand and elbow, and co-contraction between the proximal and the distal joints (Fig. 8). The decreased EMG activation levels could be attributed to the reduced spasticity that led to a reduction in the extra muscle activities [55]. The significant decrease in the EMG activation levels of the APB and the FCR-FD muscles reflected a release of muscle spasticity at the fingers and the wrist, which was manifested in decreased MAS scores in the distal joints. The reduction of excessive muscle activities of the APB and the FCR-FD suggested an improvement in muscle coordination and voluntary motor controls during grasp and release movements, which was consistent with the findings in the FMA. A significant reduction in the CI of the ECU-ED/FCR-FD and the BIC/TRI muscle pairs was observed, indicating improved coordination between the flexors and extensors at the related joints, and improved independence in muscle contraction after the training. The significant disease in the CI of the ECU-ED/BIC, FCR-FD/BIC, FCR-FD/TRI, APB/BIC muscle pairs implied a release in the muscle co-contraction between the proximal and distal joints during arm reaching/withdrawing and grasping/releasing. A significant reduction in the CI of the FCR-FD/APB muscle pair was observed, suggesting a release in muscle co-contraction between the distal joints and the thumb, which contributed to the motor improvements in hand grasping, as manifested in the increased ARAT scores. The decreased EMG activation levels and co-contractions between the antagonist muscle pairs were also related to the reductions in abnormal muscle coactivation pattern caused by obligatory synergies in many hemiparetic patients after stroke [11, 56].

#### Kinematic parameters

A significant decrease in NMUs was found after the training, indicating increased smoothness of movement (Fig. 9). Oscillatory velocity profiles with multiple peaks were observed in the performance of a limb task of a patient with stroke using a paretic limb, as opposed to the smooth bell-shaped velocity profile with one predominant peak of the nonparetic limb [50]. These peaks reflect repetitive acceleration and deceleration when performing the limb task, which was due to the limited ability of patients with stroke to produce accommodative joint torque to maintain muscle tone during multi-joint coordinated limb movements [57]. The increased smoothness suggested an improvement in fine motor control [49, 58] and improved inter-joint coordination [58], which was consistent with the observations in the cross-joint CIs. It is a common observation that stroke patients rely on compensatory strategies that involving the trunk to overcome limb impairments during arm reaching associated with distal movements [28, 59, 60]. The significant reduction in MTD revealed a reduction in compensatory trunk movements when performing the multi-joint coordinated limb task, especially during object transportation (Fig. 10). The reduced requirement of trunk involvement to assist object transportation suggested an improvement in muscle strength in the shoulder and elbow and a release of spasticity in the elbow [61].

##### Improvements in the whole upper limb

Motor improvements in the entire upper limb (including both the proximal and distal joints) were found after the training. During the training, the assistance from the EMG-driven WH-ENMS was incorporated in the coordinated tasks related to the arm reaching/withdrawing and hand open/close of the whole upper limb. Thus, the proximal joints were also practiced voluntarily, although there was no device assistance directly applied. Furthermore, the proximal improvements could also be related to the compensatory contraction of proximal upper extremity (UE) muscles during the recruitment of distal muscles in the training and the competitive interaction between distal and proximal muscles during the sequenced motion tasks [62]. It was also reported that motor improvements in both the proximal and distal joints could be obtained through physical training involving the multi-joint coordinated task [63]. Furthermore, the introduction of NMES into the distal joints could promote motor coordination between the proximal and distal joints during rehabilitation, which could facilitate the improvement in the entire upper limb [64–66].

### Independency in self-help upper limb training at home

Currently, most robotic therapies require therapists on-site, and limited techniques are available to support self-help post-stroke rehabilitation training [15, 26]. In this study, the feasibility and rehabilitation outcomes of a home-based self-help training assisted by EMG-driven WH-ENMS have been confirmed. The training setup used in the rehabilitation program and the system were easy to learn and easy to operate. All of the participants had received a pre-training tutorial and 3-session training at the laboratory before they could successfully conduct the home-based training with the prescribed training intensity and duration without any on-site professional supervision. There has been an exoskeleton robot (Myopro) developed for upper limb rehabilitation for self-help training at home for outpatients with stroke [67]. The participants had received at least 12 supervised sessions training with MyoPro (60 to 90 min per session) in the clinic to grasp device operation before the self-help training [67]. In the case of MyoPro, the rigid exoskeleton (1.8 kg, elbow–wrist–hand orthotic device) with nonnegligible weights mounted onto the paretic limb might cause incorrect alignment or migration during repeated practice [15, 68]. This additional safety concern pertaining to user-independent usage at home probably led a longer supervised training duration for the patients to gain the competency required for independent use of the device.

In the present study, the training progress of the participants was telemonitored and found to be compliant with the prescribed training intensity. In the home-based training, the variation in the training among the participants ranged from 60 to 66 min per session. It is reported that training intensity and duration are important factors for clinical improvement [12, 69]. In a study on self-administered home-based training with a passive dynamic wrist-hand orthosis accompanied by gaming exercises [70], participants were trained independently at home for six weeks and were recommended to practice for 30 min per day, six days per week. Limited motor improvement (i.e., no significant improvement in FMA) was found after the training. Besides the different effectiveness levels of the assistive devices, the inability to complete the recommended training duration was another key difficulties reported in that study [70]. A large amount of variation in training duration was observed among individuals, ranging from 2 to 60 min per day, because of lenient management and control of the training schedule (e.g., a lack of make-up sessions based on a quantitative and consistent protocol and delayed monitoring and follow-up in weeks rather than in days). Therefore, efficient monitoring of the training progress according to the prescribed rehabilitative protocol and schedule is important to ensure effective home-based self-help rehabilitation.

It also suggested that malfunctioning training systems in home-based rehabilitation should be easy to restore. Otherwise, unexpected drop-outs of recruited patients and violation of the training protocol could be encountered, as reported in a study on technology-supported home-based training [71]. In the present study, the technical problems were solved by directly replacing the malfunctioning systems efficiently with backup systems without violating the training protocol, leading to effective rehabilitation and smooth user experience. Moreover, the availability of multiple duplications of the device required a low-cost design for home-based training systems.

The compact design of the system could increase the feasibility of robot-assisted home-based training. Several existing home-based rehabilitation robots require a large physical space in the user’s living environment [16], which could be a challenge for patients living in crowded or small spaces [21, 72]. All of the participants in this study had adequate space in their house to accommodate the EMG-driven WH-ENMS due to the mobile and compact design of the system. Furthermore, the existing robotic devices are usually heavy and complex and require regular home visits for on-site installation, maintenance, and retrieval [16]. In the case of the system used in the present study, the participants could bring the system back to the laboratory for replacement or return without the need for additional home visits by the experimental operator.

The system provides flexibility in terms of training schedule; for instance, a patient can choose to use it at weekends or even at midnight, which is not possible with traditional clinical services. It was found that the preferred training time slots selected by the participants were 14:00 to 16:00 and 19:00 to 21:00. However, in traditional out-patient rehabilitation, it is difficult to accommodate all patients in these time slots. This could be one of the reasons for the low compliance with and attendance of long-term service for outpatient rehabilitation after stroke [73, 74]. Furthermore, the home-based upper limb rehabilitation of four participants in this study was not stopped during the COVID-19 pandemic, but conventional face-to-face physiotherapy and occupational therapy have been largely suspended due to social distancing restrictions worldwide. The robotic technique and the associated home-based and self-help training mode in this study provided an additional and effective option to the traditional rehabilitation to minimize the impact of physical distance control during the pandemic.

### Limitation and future work

A single-group trial was used in this study to investigate the feasibility and effectiveness of EMG-driven WH-ENMS assisted home-based self-help telerehabilitation. The results demonstrated that it was feasible for persons with chronic stroke to complete the training tasks in a home-based environment with significant motor improvements. A randomized controlled trial with a larger sample size will be conducted in future studies to compare the rehabilitation effectiveness of the EMG-driven WH-ENMS assisted telerehabilitation with the conventional treatments. The psychometric information during the home-based training has not been collected in this study. It will also be investigated by surveys in future studies.

The training task in this study is to simulate the coordination of the joints in the arm reaching out, hand open for grasping, and withdrawing in daily activities, in order to restore the ability in the performance of daily tasks to the patients with stroke. During the EMG measurement and kinematic measurement, the participant was required to perform the training task without assistance from the EMG-driven WH-ENMS to evaluate if the participants have gained the ability to perform the trained movement. However, using the training tasks in the evaluation was a limitation to investigate the generalization of the motor gains in ADLs. In the result measured by FIM, the motor improvements in the affected limb have not been translated into the functional use in ADLs for the chronic stroke in this work. A more detailed design of the evaluation on the generalization of the motor improvements in ADLs will be included in our future studies.

## Conclusion

The results of this study suggested that the home-based self-help upper limb training assisted by the EMG-driven WH-ENMS was feasible and effective for improving upper limb function after stroke. After the training program, the participants exhibited significant motor improvement in the entire upper limb. Significant improvements were found in the voluntary motor control and muscle coordination of the upper limb, the increased smoothness and reduced compensatory trunk movement during arm reaching coordinated with distal movements, and the release of muscular spasticity at the elbow, wrist and fingers. This new training mode of home-based self-help telerehabilitation could be an additional and effective option to support regular and long-term rehabilitation for outpatients with stroke.

## Supplementary Information

**Additional file 1: Appendix S1.** Part A. Evaluation of muscular coordination in the upper limb by EMG. Part B. Evaluation of movement smoothness and compensatory trunk movement.

**Additional file 2: Appendix S2.** **Table S1A.** The demographic characteristics of each patient with stroke recruited for the home-based self-help upper limb training program (n =11). **Table S1B.** The 20-session training data (including training frequency, session duration and complete wrist/hand movement cycles per session) of each participant recruited for the home-based self-help upper limb training program (n =11). **Table S1C.** The individual outcomes of clinical scores of each participant recruited for the home-based self-help upper limb training program (n =11).

## Data Availability

The datasets analyzed in the current study are not publicly available, because it has been stated in the consent approved by the Human Subjects Ethics Sub-Committee of the Hong Kong Polytechnic University that the results of the experiment may be published, but the individual results should be kept confidentially for each subject.

## Abbreviations

ADLs: Activities of daily living
VR: Virtual reality
NMES: Neuromuscular electrical stimulation
EMG: Electromyography
ENMS: Exoneuromusculoskeleton
WH-ENMS: Wrist/hand exoneuromusculoskeleton
APB: Abductor pollicis brevis
FCR: Flexor carpi radialis
FD: Flexor digitorum
FCR-FD: Flexor carpi radialis-flexor digitorum
ECU: Extensor carpi ulnaris
ED: Extensor digitorum
ECU-ED: Extensor carpi ulnaris-extensor digitorum
BIC: Biceps brachii
TRI: Triceps brachii
SD: Standard deviation
MMSE: Mini-Mental State Examination
FMA: Fugl-Meyer Assessment
ARAT: Action Research Arm Test
WMFT: Wolf Motor Function Test
FIM: Functional Independence Measurement
MAS: Modified Ashworth Score
CI: Co-contraction index
MVCs: Maximum voluntary contractions
NMUs: Number of movement units
MTD: Maximal trunk displacement
EF: Effect size
UE: Upper extremity
