# Supplementary material for: Home-based self-help telerehabilitation of the upper limb assisted by an electromyography-driven wrist/hand exoneuromusculoskeleton after stroke
Source: J Neuroeng Rehabil. 2021 Sep 15;18:137. doi: 10.1186/s12984-021-00930-3 (PMC8442816; doi:10.1186/s12984-021-00930-3)
Supplement: Supplementary file 1 — Additional file 1: Appendix S1. Part A. Evaluation of muscular coordination in the upper limb by EMG. Part B. Evaluation of movement smoothness and compensatory trunk movement. [file 12984_2021_930_MOESM1_ESM.docx]

**Appendix 1 - Part 1A**

**Evaluation of muscular coordination in the upper limb by EMG**

EMG recordings of the maximum voluntary contractions (MVCs) [57] and a bare arm test [66,76] were performed during the evaluation. EMG electrode pairs (2 × 3 cm^2^, Blue Sensor N, Ambu Inc., Ballerup, Denmark) were attached to the skin surface of the following target muscle unions or muscles of the paretic limb: abductor pollicis brevis (APB), FCR-FD muscle union, ECU-ED muscle union, biceps brachii (BIC) muscles, and triceps brachii (TRI) muscles (the configuration specified in a previous study [36] was used). The participants were required to use their paretic limbs to perform a bare arm test that was similar to the motion tasks in the training (Table 1) without any assistance from the system and complete the tasks at their natural speed, with the seating arrangement shown in Figure 5. Both the horizontal and vertical tasks were repeated thrice. There was a 2-min rest between two consecutive contractions to prevent muscle fatigue. In each task, the EMG recording was started once the participant’ hand touched the sponge and ended once all of the fingers left the sponge at the starting point. A 10-s maximum time limit was set at the end of the attempt to perform the release action during the tasks [66,77]. If the participants could not release the sponge within 10-s during the tasks, they could use their unaﬀected hands to remove the sponge. Most of the participants (n = 8) could not release the sponge by using their paretic hands due to the spasticity at the flexors and the weakness at the extensors of the distal joints [78,79]. In the post-training evaluation, two of them could release the sponge without assistance from their unaﬀected hands.

The collected EMG signals were first amplified with a gain of 1000 (amplifier: INA 333, Texas Instruments Inc., Dallas, TX, USA), band-pass filtered from 10 to 500 Hz, and then sampled at 1000 Hz for digitization for offline processing [65,77].

The EMG activation level of a muscle was calculated as follows:

$$\bar{\mathrm{EMG}}=\frac{1}{T}\int_{0}^{T} {EMG}_{i}\left( t \right)dt , \left( 1 \right)$$

where $\bar{\mathrm{EMG}}$ refers to the average EMG envelope value of muscle *i*, $\mathrm{EMG}_{i}\left( t \right)$ is the EMG envelope signal obtained after normalization with respect to the EMG MVC value of the muscle, and *T* is the length of the signal.

The CI between a pair of muscles can be expressed as follows:

$$CI=\frac{1}{T}\int_{0}^{T} A_{ij}\left( t \right)dt , (2)$$

where A_ij_(t) is the overlapping activity of EMG linear envelopes for muscles *i* and *j*, and *T* is the length of the signal. An increase in the CI value represents an increased co-contraction of a muscle pair (broadened overlapping area), and a decrease in the CI value indicates a decreased co-contraction of a muscle pair (reduced overlapping area).

**Appendix 1 - Part 1B**

**Evaluation of movement smoothness and compensatory trunk movement**

During motion capturing, the same bare arm test was conducted. The participants were required to repeat both the horizontal and the vertical tasks thrice (Table 1). There was a 2-min break between two consecutive contractions to prevent muscle fatigue. A total of 25 spherical reflective markers (14-mm diameter for each) were attached to the skin of the upper limb and the body trunk in accordance with the upper limb model of the BodyBuilder model (Vicon Motion Systems, Oxford, UK [80]) (Figure 6). The marker positions were captured using an eight-camera motion system (Vicon Motion Systems, Oxford, UK) at a sampling frequency of 250 Hz. A Vicon Workstation (Vicon Motion Systems, Oxford, UK) with three-dimensional reconstruction software (Vicon Nexus and BodyBuilder, Oxford, UK) was used to anatomically label, filter, and apply the upper limb model [47,48,80]. The positions and velocities of the markers during the motion were thus obtained in all trials [81].

The tangential velocity profile of the hand marker (placed on the metacarpophalangeal joint of the middle finger) in the transporting phases of the tasks (Table 1) was used to compute the NMUs [49]. In a trial, the recording started once the hand of a participant left the target position (i.e., table surface or the top of the shelf) and ended once the hand of the participant touched another target position (i.e., table surface or the top of the shelf).

The NMUs can be expressed as follows:

$$\left\{ \begin{aligned} NMUs=\sum Count \\ Count=\left\{ \begin{aligned} 1， if max-min > 0.15(MAX) \\ 0，else , \end{aligned} \right. \end{aligned} \right. (3)$$

where $NMUs$ is the cumulative counted number of a signified movement unit $Count$. A movement unit was signified once an increase had emerged between the adjacent minimum velocity$min$ and maximum velocity $max$ and had exceeded a threshold level that was 15% of the maximal velocity $MAX$ in the trial. NMUs has been used to quantify movement smoothness [49]. An increase in NMUs indicates decreased movement smoothness.

The MTD can be formulated as follows:

$$\left\{ \begin{aligned} MTD=MAX \left\{ D(t\left. ) \right\} \right. \\ D\left( t \right)=\sqrt{\left\{ (x_{t}-x_{0})^{2} \right.+(y_{t}-y_{0})^{2}+{(z}_{t}-z_{0}\left. )^{2} \right\}} , \end{aligned} \right. (4)$$

where MTD refers to the maximal displacement $MAX \left\{ D(t\left. ) \right\} \right.$of the thorax marker (placed on the jugular notch where the clavicles meet the sternum) from the initial position during the entire motion task [49]. Each calculated displacement $D\left( t \right)$ was the distance between the initial position ($x_{0}, y_{0}, z_{0}$) and each recorded position ($x_{t}, y_{t}, z_{t}$) in a trial [82]. The recording of the marker position was started when a participant touched the sponge and ended when the participant released the sponge at the starting point (all of the fingers left the sponge). MTD has been used to quantify compensatory trunk movements during limb movements, where a decrease in MTD indicates a reduction in compensatory trunk movement when the limb tasks are being performed [49]. The MTD can also be used to determine whether the smoother movement is the result of the motor function recovery or the compensatory strategies adopted by participants [58]. A 20-min break was provided between two consecutive measurements to avoid muscle fatigue.
