# Supplementary material for: Home-based self-help telerehabilitation of the upper limb assisted by an electromyography-driven wrist/hand exoneuromusculoskeleton after stroke
Source: J Neuroeng Rehabil. 2021 Sep 15;18:137. doi: 10.1186/s12984-021-00930-3 (PMC8442816; doi:10.1186/s12984-021-00930-3)
Supplement: Supplementary file 2 — Additional file 2: Appendix S2.Table S1A. The demographic characteristics of each patient with stroke recruited for the home-based self-help upper limb training program (n =11). Table S1B. The 20-session training data (including training frequency, session duration and complete wrist/hand movement cycles per session) of each participant recruited for the home-based self-help upper limb training program (n =11). Table S1C. The individual outcomes of clinical scores of each participant recruited for the home-based self-help upper limb training program (n =11). [file 12984_2021_930_MOESM2_ESM.docx]

| **Subjects No.** | **Gender** | **Stroke Types** | **Side of Hemiparesis** | **Age (years)** | **Years after onset of stroke** |
| --- | --- | --- | --- | --- | --- |
| 1 | Female | Ischemic | Left | 57 | 6 |
| 2 | Male | Ischemic | Right | 72 | 23 |
| 3 | Female | Hemorrhagic | Right | 35 | 8 |
| 4 | Female | Hemorrhagic | Right | 40 | 11 |
| 5 | Male | Ischemic | Left | 64 | 15 |
| 6 | Female | Hemorrhagic | Left | 57 | 40 |
| 7 | Female | Hemorrhagic | Left | 65 | 3 |
| 8 | Male | Ischemic | Left | 69 | 10 |
| 9 | Male | Ischemic | Left | 73 | 19 |
| 10 | Male | Hemorrhagic | Left | 38 | 2 |
| 11 | Female | Ischemic | Left | 64 | 10 |

Table S1A. The demographic characteristics of each patient with stroke recruited for the home-based self-help upper limb training program (n =11).

| **Subjects No.** | | **Frequency (session/week)** | **Duration**  **(min/session)** | **Complete movement cycles (cycle/session)** |
| --- | --- | --- | --- | --- |
|  |  |  | **Mean ± SD** | |
| 1 |  | 3 | 61.6±1.13 | 111±8.99 |
| 2 |  | 5 | 64.1±1.54 | 115±10.3 |
| 3 |  | 4 | 63.8±1.46 | 121±6.91 |
| 4 |  | 4 | 63.4±1.79 | 116±8.04 |
| 5 |  | 3 | 62.5±2.03 | 113±7.86 |
| 6 |  | 4 | 62.7±2.00 | 115±8.85 |
| 7 |  | 3 | 63.0±1.86 | 118±7.70 |
| 8 |  | 4 | 62.9±2.14 | 117±7.33 |
| 9 |  | 5 | 63.7±1.72 | 119±8.83 |
| 10 |  | 3 | 63.4±1.66 | 118±6.95 |
| 11 |  | 3 | 61.9±1.62 | 115±7.94 |

Table S1B. The 20-session training data (including training frequency, session duration and complete wrist/hand movement cycles per session) of each participant recruited for the home-based self-help upper limb training program (n =11).

| **Subjects No.** |  | **Training outcome (**$+/-$**)** | | | | | | | | | |
| --- | --- | --- | --- | --- | --- | --- | --- | --- | --- | --- | --- |
|  |  | **FMA** | | | **ARAT** | **WMFT** | | **FIM** | **MAS** | | |
|  |  | Full Score | Shoulder/Elbow | Wrist/Hand |  | Score | Time (s) |  | Elbow | Wrist | Finger |
| 1 |  | $+$5 | $+$1 | $+$4 | $+$7 | $+$3 | 0.1 | 0 | 0 | –1 | –1 |
| 2 |  | $+$7 | $+$6 | $+$1 | $+$2 | $+$1 | –0.7 | 0 | 0 | 0 | –0.4 |
| 3 |  | $+$15 | $+$6 | $+$9 | $+$5 | $+$16 | –19.6 | $+$1 | –2 | –2 | –1 |
| 4 |  | $+$24 | $+$14 | $+$10 | $+$5 | $+$13 | –12.3 | 0 | –2 | –3 | –1 |
| 5 |  | $+$7 | $+$5 | $+$2 | $+$16 | $+$3 | –2.4 | 0 | –1 | –1 | 0 |
| 6 |  | $+$14 | $+$11 | $+$3 | $+$2 | $+$5 | –0.3 | 0 | 0 | 0 | 0 |
| 7 |  | $+$18 | $+$17 | $+$1 | $+$9 | 0 | –5.5 | 0 | –0.6 | –0.4 | 0 |
| 8 |  | $+$12 | $+$10 | $+$2 | $+$13 | $+$12 | –20.8 | 0 | 0 | 0 | 0 |
| 9 |  | $+$5 | $+$1 | $+$4 | $+$17 | $+$14 | –1.4 | 0 | 0 | 0 | –1 |
| 10 |  | $+$7 | 0 | $+$7 | $+$2 | $+$3 | –1.5 | 0 | –1 | 0 | 0 |
| 11 |  | $+$9 | $+$7 | $+$2 | $+$4 | $+$4 | –0.5 | 0 | –1 | –1 | –2 |

Table S1C. The individual outcomes of clinical scores of each participant recruited for the home-based self-help upper limb training program (n =11).
